# Unraveling Human AQP5-PIP Molecular Interaction and Effect on AQP5 Salivary Glands Localization in SS Patients

**DOI:** 10.3390/cells10082108

**Published:** 2021-08-17

**Authors:** Clara Chivasso, Veronika Nesverova, Michael Järvå, Anne Blanchard, Kristie L Rose, Fredrik Kryh Öberg, Zhen Wang, Maud Martin, Florent Lhotellerie, Egor Zindy, Bruna Junqueira, Karelle Leroy, Benoit Vanhollebeke, Valérie Delforge, Nargis Bolaky, Jason Perret, Muhammad Shahnawaz Soyfoo, Stefania Moscato, Chiara Baldini, François Chaumont, Letizia Mattii, Kevin L Schey, Yvonne Myal, Susanna Törnroth-Horsefield, Christine Delporte

**Affiliations:** 1 Laboratory of Pathophysiological and Nutritional Biochemistry, Université Libre de Bruxelles, 1070 Brussels, Belgium; clara.chivasso@ulb.be (C.C.); Florent.Lhotellerie@ulb.be (F.L.); Valerie.Delforge@ulb.be (V.D.); Nargis.Bolaky@ulb.ac.be (N.B.); Jason.Perret@ulb.be (J.P.); 2 Division of Biochemistry and Structural Biology, Lund University, 221 00 Lund, Sweden; verca.nesver@gmail.com (V.N.); susanna.horsefield@biochemistry.lu.se (S.T.-H.); 3 Department of Chemistry and Molecular Biology, University of Gothenburg, 907 36 Umeå, Sweden; michael.jarva@umu.se (M.J.); fredrik.kryh.oberg@gmail.com (F.K.Ö.); 4 Department of Pathology, University of Manitoba, Winnipeg, MB R3E 0T5, Canada; aaablanch@gmail.com (A.B.); Yvonne.Myal@umanitoba.ca (Y.M.); 5 Department of Biochemistry, Vanderbilt University School of Medicine, Nashville, TN 37240, USA; kristie.rose@Vanderbilt.Edu (K.L.R.); zhen.wang@Vanderbilt.Edu (Z.W.); k.schey@Vanderbilt.Edu (K.L.S.); 6 Laboratory of Neurovascular Signaling, Université Libre de Bruxelles, 6041 Gosselies, Belgium; maud.martin@ulb.be (M.M.); Benoit.Vanhollebeke@ulb.be (B.V.); 7 Multimodal Image Processing, Center for Microscopy and Molecular Imaging (CMMI), 6041 Gosselies, Belgium; egor.zindy@ulb.be; 8 Louvain Institute of Biomolecular Science and Technology, UCLouvain, 1348 Louvain-la Neuve, Belgium; bruna.teodoro@uclouvain.be (B.J.); francois.chaumont@uclouvain.be (F.C.); 9 Laboratory of Histology, Neuroanatomy and Neuropathology, UNI (ULB Neuroscience Institute), Faculty of Medicine, Université Libre de Bruxelles, 1070 Brussels, Belgium; kleroy@ulb.be; 10 Department of Rheumatology, Erasme Hospital, Université Libre de Bruxelles, 1070 Brussels, Belgium; muhammad.shah.soyfoo@ulb.be; 11 Department of Clinical and Experimental Medicine, University of Pisa, 56126 Pisa, Italy; stefania.moscato@unipi.it (S.M.); chiara.baldini74@gmail.com (C.B.); letizia.mattii@med.unipi.it (L.M.); 12 Research Institute for Oncology and Hematology (RIOH), CancerCare Manitoba, Winnipeg, MB R3E 0V9, Canada

**Keywords:** aquaporin-5, Sjögren’s syndrome, prolactin-inducible protein, salivary gland

## Abstract

Saliva secretion requires effective translocation of aquaporin 5 (AQP5) water channel to the salivary glands (SGs) acinar apical membrane. Patients with Sjögren’s syndrome (SS) display abnormal AQP5 localization within acinar cells from SGs that correlate with sicca manifestation and glands hypofunction. Several proteins such as Prolactin-inducible protein (PIP) may regulate AQP5 trafficking as observed in lacrimal glands from mice. However, the role of the AQP5-PIP complex remains poorly understood. In the present study, we show that PIP interacts with AQP5 in vitro and in mice as well as in human SGs and that PIP misexpression correlates with an altered AQP5 distribution at the acinar apical membrane in PIP knockout mice and SS hMSG. Furthermore, our data show that the protein-protein interaction involves the AQP5 C-terminus and the N-terminal of PIP (one molecule of PIP per AQP5 tetramer). In conclusion, our findings highlight for the first time the role of PIP as a protein controlling AQP5 localization in human salivary glands but extend beyond due to the PIP-AQP5 interaction described in lung and breast cancers.

## 1. Introduction

**Figure 1:**
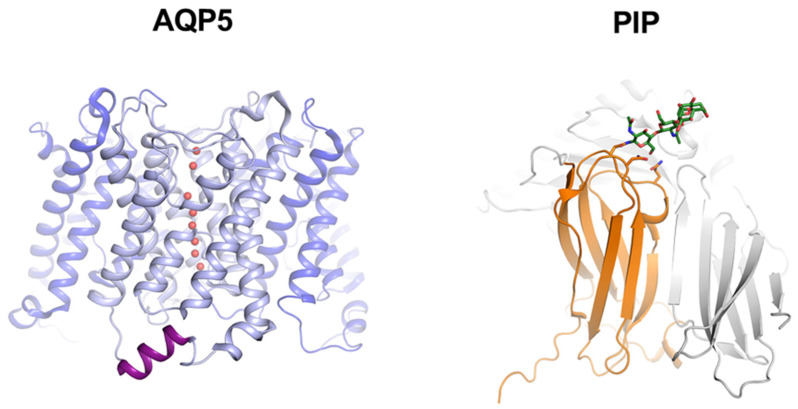
**Left**: crystal structure of the human AQP5 tetramer (PDB code 3D9S) with one monomer highlighted in light blue and water molecules in red. The C-terminal helix that is known to be involved in protein-protein interactions in other AQPs is highlighted in purple. The last 21 amino acids were not resolved in the crystal structure and are therefore not shown. **Right**: crystal structure of human PIP (orange) in complex with the Zinc α2-glycoprotein (ZAG, grey) (PDB code 3ES6). A single glycosylation chain on Asn77 in PIP is shown in green.

Sjögren’s syndrome (SS) is a chronic autoimmune disease characterized by salivary and lacrimal hyposecretion, lymphocytic infiltration, and glandular destruction [1]. Aquaporin-5 (AQP5) is a member of the aquaporin (AQP) family of membrane-bound water channels ensuring transmembrane water passage according to osmotic gradient [2]. Functional AQP5 is a homotetramer, each monomer containing six transmembrane helices and two loops buried in the membrane that create a water-specific pore (Figure 1) [3]. It is regulated by trafficking to the plasma membrane, thereby controlling transmembrane water permeability. Upon nerve stimulation of salivary glands (SGs), AQP5 translocation to the acinar apical plasma membrane plays a key role in saliva fluid secretion by facilitating water passage [4,5]. In lacrimal glands (LGs) and SGs acinar cells from SS patients, AQP5 is aberrantly located in the cytoplasm or basolateral membrane, whereas in normal tissues AQP5 is primarily located in the apical membrane [6,7,8]. Recently, defect in AQP5 localization in LGs has been linked to the loss of prolactin-inducible protein (PIP) expression and AQP5-PIP interaction in the non-obese diabetic (NOD) mouse model of SS [9]. Moreover, the role of PIP in SS pathogenesis was heightened by studies showing PIP downregulation in rabbit tears following the induction of inflammation in lacrimal glands [10] and lower levels of PIP in saliva and human minor salivary glands (hMSG) from SS patients [11,12].

PIP is a glycosylated protein expressed in various human tissues [13] including LGs and SGs [14,15]. Its structure consists of seven antiparallel β-strands that pack into two β-sheets with a cluster of hydrophobic residues between the sheets (Figure 1) [16]. PIP glycosylation pattern depends on tissue and pathological condition and is involved in its ability to interact with other proteins such as CD4 and fibronectin [17]. PIP knockout mouse model shows a normal development and fertility, but several abnormal phenotypes have been described such as enlarged lymph nodes around the parotid glands and thymic medulla and an important decrease in the CD4+ T cell number and differentiation [18].

Our study aimed to deepen our understanding of AQP5-PIP interaction and its role in SS abnormal AQP5 localization observed in human MSG. To achieve this goal, AQP5-PIP interaction was studied in a human cell line and in vivo mouse model and finally in the human pathological setting of SS in patient samples.

## 2. Materials and Methods

### 2.1. Plasmid Preparation

PCR was carried out on cDNA from human lung to amplify AQP5 coding sequence without or with Kozak a consensus (Koz), using Phusion U Master Mix (Thermo-Fisher Scientific, Waltham, MA, USA). The AQP5 amplicon without a Kozak consensus was cloned downstream from an HA-tag (human influenza hemagglutinin) in pcDNA3.1 to generate the HA-AQP5 plasmid. HA-CT plasmid is the empty vector containing the HA-tag in pcDNA3.1. The AQP5 amplicon with a Kozak consensus was cloned in pcDNA3.1 to generate the Koz-AQP5 plasmid. PIP-encoding plasmid (pEZ-M94 vector) came from GeneCopoeia (Rockville, MD, USA).

### 2.2. cRNA Preparation and Xenopus leavis Swell Assay

HA-AQP5 and Koz-AQP5 plasmid DNA were linearized and then transcribed in vitro with T7 RNA polymerase using mMessage mMachine kit (Thermo-Fisher Scientific). cRNA quality was verified using Experion (Biorad, Hercules, CA, USA). *Xenopus laevis* oocytes (15 per group; 2 experiments) were injected with 50 nL of sterile water (control), 50 nL of 100 ng/µL cRNA of HA-AQP5 or koz-AQP5 (5 ng of cRNA/oocyte) (EcoCyte Bioscience, Dortmund, Germany). Oocytes that died prior to the end of the experiments were excluded from the analysis (6 oocytes injected with Koz-AQP5; 2 oocytes injected with HA-AQP5). The sample size was determined based on P_f_ variability obtained previously [19]. After two days incubation in Barth’s medium (88 mM NaCl, 1 mM KCl, 2.4 mM NaHCO_3_, 10 mM HEPES-NaOH, 0.33 mM Ca(NO_3_)_2_, 0.41 mM CaCl_2_, 0.82 mM MgSO_4_, pH 7.4), the oocytes were subjected to an hypotonic challenge in 5-fold-diluted Barth’s medium at room temperature. The increase in oocyte volume was recorded every 5 s for 70 s using a microscope linked to a camera. The osmotic water permeability coefficient (P_f_) was calculated using the equation P_f_ = V0[d(V/V0)/dt)/[A0 × Vw (Osmoout-Osmoin)] as described previously [19]. V0 is the initial oocyte volume, V is the final oocyte volume following hypotonic perfusion, A0 is the initial oocyte area, Vw is the partial molar volume of water which corresponds to 18 cm^3^/mol, and (Osmoout-Osmoin) is the osmotic gradient.

### 2.3. Isolation of Oocytes Membrane

Microinjected oocytes (12 per group) were homogenized in 10 mM KH_2_PO_4_, 5 mM EDTA, 5 mM EGTA, and protease inhibitors. The homogenate was centrifuged at 4 °C for 5 min at 500× *g* and then the supernatant was centrifuged for 30 min at 16,000× *g*. The pellet containing the oocytes membranes was washed with homogenization buffer and resuspended in 30 µL of sterile water containing protease inhibitors. Western blotting was performed as described below.

### 2.4. Cell Culture and Transfection

Normal SG-SV40 transformed-squamous cells resembling acinar cells [20] (NS-SV-AC cells; a generous gift from Prof. M. Azuma, Second Department of Oral and Maxillofacial Surgery, Tokushima University School of Dentistry), were grown in DMEM-HamF12 medium containing 5% heat-inactivated fetal calf serum, 100 UI/mL streptomycin-penicillin and 4 mM glutamine (Thermo-Fisher Scientific) and passaged twice a week. The NS-SV-AC cell line has been tested for the absence of mycoplasma contamination using the LookOut^®^ Mycoplasma PCR Detection Kit (Sigma-Aldrich, St. Louis, MO, USA). Short tandem repeat (STR) DNA profile confirmed the cell line identity (European Collection for Authenticated Cell cultures, Public Health England, England). As NS-SV-AC cells, did not express detectably AQP5 and PIP, they were transfected by electroporation (270 V, 700 μF, using a Gene Pulser II System (Bio-Rad, Hercules, CA, USA)) with a mixture of 8 µg of the two plasmids (each at 4 µg).

### 2.5. NS-SV-AC Proximity Ligation Assay

Proximity ligation assays (PLA) were performed using Duolink kit (Sigma-Aldrich, St. Louis, MO, USA) on transfected NS-SV-AC cells seeded on Millicell EZ SLIDE 8-well glass (Millipore, Burlington, MA, USA) and fixed 48 h post-transfection with methanol for 15 min at −20 °C. Mouse anti-HA-tag (Proteintech, Rosemont, IL, USA) and rabbit anti-PIP (Novus Biological, Littleton, CO, USA) were used at 1:100 and 1:200 dilutions, respectively. Negative controls were performed in the absence of one or both antibodies. Z-stack images were acquired using a confocal microscope (LSM-710) with an ×63/1.4 PlanApochromat lens (Zeiss, Oberkochen, Germany).

### 2.6. PIP Cloning, Expression, Purification, and Deglycosylation

The PIP sequence (P12273, residues 29–146) containing codons for a thrombin-cleavable 6× His-tag on the C-terminus was cloned into the plasmid pPICZαA. This plasmid was transformed into the yeast *Pichia pastoris*. High zeocine selection was used to isolate a clone with multiple copies of the plasmid and therefore potentially higher protein yield. The cells were grown in Basal Salt Medium in a fermenter (Belach Bioteknik) and PIP was expressed upon induction with methanol for 46 h. Because PIP was targeted for secretion into the media using the secretion signal sequence present on the plasmid, the cell culture supernatant was filtered and concentrated using a Vivacell 100, 5 kDa MWCO (Sartorius, Göttingen, Germany). The pH was adjusted to 8 using sodium hydroxide to precipitate some salts present in the growth media. The sample was then filtered again prior to the addition of imidazole to a final concentration of 50 mM and loaded on a HisTrap HP 5 mL column (GE Healthcare, Little Chalfont, UK) equilibrated with a buffer A (50 mM Tris-HCl pH 8, 25 mM NaCl, 5% glycerol, 5 mM DTT) containing 50 mM imidazole. The protein was eluted by increasing the imidazole concentration to 7 mM. Relevant fractions were pooled, concentrated using a 10 kDa MWCO spin concentrator, and loaded onto a size exclusion column (Superdex 200 10/300 GL, GE Healthcare) equilibrated with buffer A. The typical protein yield was 4 mg/L of media. The glycans were removed from PIP by treatment with PNGase F (NEB) under denaturing conditions according to the manufacturer’s protocol. A control sample was treated in the same way but without an enzyme. The samples were analyzed on SDS-PAGE with SimplyBlue SafeStain (Thermo-Fisher Scientific) and WB using anti-His primary antibody (Clontech, Mountain View, CA, USA). Pro-X Emerald 300 glycoprotein gel kit (Thermo-Fisher Scientific) was used to visualize only glycosylated proteins on SDS-PAGE. A CandyCane glycoprotein ladder (Thermo-Fisher Scientific) was loaded to estimate molecular weights of the protein bands and positive control.

### 2.7. AQP5 Expression, Purification

The generation of the AQP5 constructs, membrane preparation, and protein purification have been previously described for a full-length construct with His-tag and full-length construct without His-tag [21]. The untagged truncated constructs N228Stop and T242Stop were generated using site-directed mutagenesis. Briefly, all constructs were cloned into a pPICZB plasmid and the protein was expressed in Pichia pastoris X-33 cells using a fermenter. The cells were broken in a French press or a Bead beater and the cell debris was spun down at 16,000× *g*. The membranes were isolated by ultracentrifugation at 100,000× *g* and washed with a buffer containing 4 M urea, 5 mM Tris-HCl pH 9.5, 2 mM EDTA, and 2 mM EGTA. The washed membranes were spun as before and then washed again, this time with 20 mM sodium hydroxide. After a final ultracentrifugation step, the resulting double-washed membrane pellet was resuspended in a buffer containing 20 mM HEPES pH 7.8, 50 mM NaCl and 10% glycerol and kept at −80 °C until further use.

AQP5 was solubilized in 3% n-Nonyl-β-D-Glucopyranoside (NG, Anatrace, Maumee, OH, USA) for 1h at room temperature. Non-solubilized material was separated by ultracentrifugation. The pH of the supernatant was adjusted to 6 for full-length constructs and to 7 for the truncated mutants. The sample was loaded on Resource S cation exchange column (GE Healthcare, Chicago, IL, USA) and eluted with a NaCl gradient (15 mM–1 M). All buffers contained 0.4% NG and either 20 mM MES pH 6 or 20 mM MOPS pH 7. Fractions containing AQP5 were pooled, concentrated using a 50 kDa MWCO spin concentrator and loaded onto a Superdex 200 10/300 column, equilibrated with 20mM Tris-HCl pH 7.4, 100 mM NaCl and 0.4% NG. AQP5 is typically eluted as a symmetric peak at around 13 mL.

### 2.8. Co-Elution Assay

First, a HisTrap HP (GE Healthcare) 1 mL column was equilibrated with a buffer containing 20 mM phosphate buffer pH 7.5, 300 mM NaCl, 10 mM imidazole, 2 mM TCEP, and 0.4% NG. Purified PIP was bound to the column and all unbound PIP was washed off with the buffer. Next, full-length AQP5 without His-tag was looped over the column overnight after which any unbound AQP5 was washed. Proteins were eluted with 300 mM (sample E1) and 400 mM (sample E2) imidazole. The peak fractions were concentrated and analyzed on SDS-PAGE with SYPRO Ruby total protein staining and glycosylation specific staining. A WB was performed using antibodies against the 6× His-tag on PIP and the signal was detected using the ONE-HOUR western Detection Kit (GenScript, Piscataway, NJ, USA).

### 2.9. Microscale Thermophoresis

Purified PIP was fluorescently labeled with an amine-reactive dye using the Monolith NT^ΤΜ^ Protein RED-NHS Labeling Kit including buffer exchange. The labeled PIP was loaded onto a PD-10 desalting column (GE Healthcare) and eluted into the MST buffer (20 mM phosphate buffer pH 7.5, 100 mM NaCl, 0.8% NG).

For the microscale thermophoresis (MST) experiment a 1:1 dilution series of AQP5 in MST buffer was prepared. Next, the labeled PIP was added (final concentration 42 nM) and the samples were loaded into standard glass capillaries and analyzed for thermophoresis in the Monolith NT.115 instrument (NanoTemper Technologies, Munich, Germany). Full-length His-tagged AQP5, as well as untagged N228Stop and T242Stop, were individually tested for binding to PIP. The LED power was set to 25% and the MST power to 40%.

The MST data obtained from each triplicate was averaged. Since the concentration of labeled PIP was well below the K_d_, the concentration of added AQP5 and the concentration of unbound AQP5 were almost the same. The data could be fitted with the Hill equation:

$$y=F_{\mathrm{UB}}+\frac{F_{B}-F_{\mathrm{UB}}}{1+{\left(\frac{K_{d}}{\left[\mathrm{AQP}5\right]}\right)}^{n}}$$

where FB: signal from the bound state; FUB: signal from the unbound state; K_d_: dissociation constant; [AQP5]: total monomeric AQP5 concentration; n: Hill coefficient.

In the fit, each point was weighted by the standard deviation of averaging. The standard errors of the mean (S.E.M.) for the calculated K_d_ and the Hill coefficient were derived from the variance of the fit. The fitting was performed using the non-linear least-squares method in the Python library, SciPy.

The MST stoichiometry experiments were performed at a 5 μM concentration of labeled PIP, which is well above the K_d_. The LED power and MST settings were 4% and 60%. When all binding sites on PIP were occupied, the data showed a typical saturation kink and an excess of AQP5 would not cause any change in ΔF. Ratios of AQP5 (monomer): PIP between 0 and 10 were probed. The experiment was done twice, and the data were averaged. Two linear equations were fit to the points in a way so that the total error from the fitting was as low as possible. The intersection of these two lines marks the saturation kink which could be calculated by

$$x=\frac{b2-b1}{m1-m2}$$

where m: slope; b: y-intercept in the linear equation y = mx + b. To calculate the S.E.M. for the intercept of the x-coordinate, the correlated uncertainty propagation method was used.

### 2.10. Immunoprecipitation and Western Blotting

SMGs and PGs dissected from C57Bl6 mice (Janvier Labs, Le Genest-Saint-Isle, France) were homogenized using an IKA ultra Turrax (Staufen, Germany) in ice-cold homogenization buffer (180 mM Tris containing 0.1 µM CaCl_2_, 0.8 mM MgCl_2_, 0.01% SDS, 0.05% sodium deoxycholate, 0.1% Triton X-100, 0.5 mM NaF, 0.01 mM sodium vanadate and cOmplete™ EDTA-free protease inhibitor cocktail (one tablet per 10 mL; Sigma-Aldrich, St. Louis, MO, USA), pH 7.2). Homogenates were mixed for 30 min at 4 °C using a rotating shaker and centrifuged at 17,000× *g* for 20 min at 4 °C. Supernatants were collected and total protein content was determined using a Pierce BCA protein assay (Thermo-Fisher Scientific). SMG and PG total proteins were incubated overnight at 4 °C in the absence (negative control) or presence of rabbit anti-AQP5 (1 µL per 800 µg of protein; EMD Millipore, Burlington, MA, USA), followed by incubation with protein A-coated Sepharose beads for 1 h at 4 °C. Additional negative control was performed in the presence of beads and antibodies but the absence of SM or P proteins. Beads were washed 3 times with homogenization buffer and bound proteins were eluted with 20 µL of Laemmli buffer containing 10 mg/mL dithiothreitol following a 30 min incubation at 37 °C and centrifugation at 17,000× *g* for 5 min at room temperature.

Immunoprecipitated proteins and total input proteins were separated using a 12% SDS-PAGE Tris-Glycine gel and electrotransferred to PVDF membrane (Invitrogen, Carlsbad, CA, USA). PVDF membrane was blocked for 60 min at room temperature in 5% milk diluted into PBS-0.1% Tween, then incubated overnight at 4 °C with rabbit anti-AQP5 antibody (EMD Millipore, Burlington, MA, USA) diluted at 1:1000 in 5% milk PBS-0.1% Tween. PVDF membrane was incubated for 1 h at room temperature with anti-rabbit HRP-conjugated antibody diluted at 1:3000 (Cell Signaling, Danvers, MA, USA). Immunoblots were revealed by chemiluminescence (PerkinElmer; Waltham, MA, USA) using Kodak X-omat blue films.

### 2.11. In-Gel Trypsin Digestion of Immunoprecipitated Proteins and Liquid Chromatography/Electrospray Ionization MS/MS Analysis

Mouse SG immunoprecipitated proteins were separated with SDS-PAGE using a 4–12% NuPAGE Novex Bis-Tris gel (Thermo-Fisher Scientific). The gel was stained with Coomassie blue to allow the excision of visible gel bands. Gel bands were destained prior to in-gel trypsin digestion, as previously described [22]. Tryptic peptides were separated by one-dimensional liquid chromatography or multidimensional protein identification technology (MudPIT) and data were analyzed as previously described [22].

### 2.12. PIP Knockout Mice

PIP knockout mice (PIP^−/−^) were described previously [18]. Ten-week-old homozygous male and female PIP^−/−^ mice (backcrossed > 9 generations in CD1 background and wild-type (PIP^+/+^) CD1 mice were obtained from in-house breeding colony, housed and maintained in groups a pathogen-free facility (Central Animal Care Services, University of Manitoba). Animal studies were approved by the University of Manitoba Animal Care and Use Committee, in agreement with the Canadian Council on Animal Care guidelines. The male and female PIP^−/−^ mice groups were compared with their corresponding sex PIP^+/+^ mice group.

### 2.13. Mice SGs Immunohistochemistry

PIP^+/+^ and PIP^−/−^ mouse SG (5 mice per group, 1 experiment, 20 mice in total) were fixed in 4% buffered formaldehyde, paraffin-embedded, and sectioned (4 µm-thick). Two PGs could not be analyzed due to poor tissue dissection. SG sections were incubated overnight at 4 °C with rabbit anti-AQP5 antibodies (EMD Millipore, Burlington, MA, USA). The bound anti-AQP5 antibody was revealed by anti-rabbit gamma globulin (Nordic, Tilburg, The Netherlands) and peroxidase-anti-peroxidase complex (Dako, Glostrup, Denmark). The fixed peroxidase was visualized using diaminobenzidine (Dako, Glostrup, Denmark). As a negative control, SG sections were incubated with secondary antibodies alone. A counter coloration was performed with hematoxylin. Images were captured at 40× and 100× magnification using a light microscope (BX43, Olympus, Düsseldorf, Germany) and an SC50 digital camera (Olympus).

### 2.14. Human Minor SG Samples

Human minor SG biopsies paraffin blocs (Erasme Hospital Biobank, Brussels, Belgium; BE_BERA1; Biobanque Hôpital Erasme-ULB (BERA); BE_NBWB1; Biothèque Wallonie Bruxelles (BWB); BBMRI-ERIC) were sectioned by Diapath (part of the Center for Microscopy and Molecular Imaging (CMMI). All patients with SS (n = 22; 61 ± 3 years old) fulfilled the American College of Rheumatology (ACR)/European League against Rheumatism (EULAR) classification criteria for primary Sjögren’s syndrome [23]. Control patients with non-specific sialoadenitis were used as negative controls (n = 21; 69 ± 2 years old). The study was conducted on human residual material. Patients did not oppose its use for research purposes. Therefore, compliant with Belgian law, this study did not require informed consent. The study using hMSG biopsies received approval from the ULB Erasme Hospital ethics committee (P2016/299 and P2020/351).

### 2.15. hMSG Proximity Ligation Assay

PLA was performed on paraffin-embedded SG sections. Rabbit anti-AQP5 (EMD Millipore, Burlington, MA, USA) and mouse anti-PIP (Abcam, Cambridge, UK) were used at a 1:100 dilution. Negative controls were performed in the absence of one or both antibodies. Z-stack images were acquired using a confocal microscope (LSM-710) with an ×63/1.4 PlanApochromat lens (Zeiss, Oberkochen, Germany). Z-stacks were processed in FIJI (Fiji Is Just ImageJ, University of Wisconsin-Madison, Madison, WI, USA) [24], and CellProfiler (Carpenter Lab at the Broad Institute of Harvard and MIT, Cambridge, MA, USA). In FIJI, the acquired Z stacks were max-projected in the Z-axis, and each acinus of interest was manually delineated with the polygon tool. Weak background fluorescence from the AQP5 probe was used to reveal the acini structures and to guide the delineation process. For each max-projected stack, these polygons were converted into a binary mask image and added to the existing Texas Red and DAPI channels. These 2-D, 3-channel images were subsequently processed in CellProfiler as follows: Nuclei detected in the DAPI channel and inside a masked acinus were expanded into cells using a morphological dilation operation, until they touched or reached the edge of their acinus mask. In each cell thus defined, the number of detected AQP5-PIP spots was recorded for further analysis. Additional metrics, such as cell area, number of nuclei per acinus, and distance of the probe spots to their respective acinus edge were calculated and recorded for each image.

### 2.16. Double Immunofluorescence

Paraffin-embedded SG sections were incubated overnight at 4 °C with rabbit anti-AQP5 (EMD Millipore, Burlington, MA, USA) and mouse anti-PIP (Abcam, Cambridge, UK) antibodies. The anti-rabbit-AQP5 antibody signal was revealed using a biotinylated goat anti-rabbit antibody and streptavidin-conjugated Alexa 594 and the anti-mouse-PIP antibody was revealed with a goat anti-mouse, a mouse peroxidase-anti-peroxidase complex (Jackson ImmunoResearch, Ely, UK), and a FITC-conjugated Tyramide (Thermo-Fisher Scientific). Images were taken using Leica DM 2000 microscope. Immunofluorescence positivity was quantified on the images captured at 20×. One microscopic field, generally containing the whole section, was analyzed for each sample. In every microscopic field, only parenchyma tissue containing acini was selected and the reacting surfaces were quantified with CellSens Imaging Software (Olympus, Düsseldorf, Germany). The medium color threshold was evaluated on negative controls. Image analysis was performed considering the percentage of the reacting area and the respective level of pixel color intensity per field. AQP5 or PIP reactivity degree was calculated as the product between the average positive area percentage and the mean value of pixel color intensity per microscopic field.

### 2.17. Statistical Analyses

Shapiro Wilkinson test (test of normality), Student’s *t*-test, ANOVA, or Mann Whitney *u*-test were performed using IBM SPSS Statistics 25. Data are expressed as mean ± S.E.M. of n experiments. Data are considered significant when *p* < 0.05.

## 3. Results

### 3.1. In Vitro Assessment of Human AQP5 and PIP Protein-Protein Interaction in NS-SV-AC Cells

**Figure 2:**
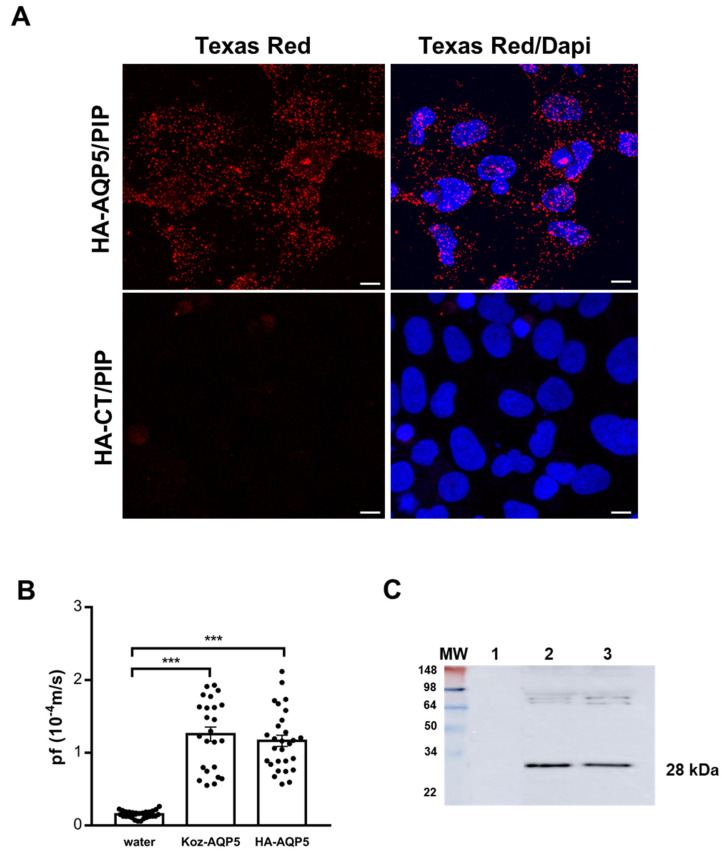
(**A**) PLA performed on transfected NS-SV-AC cells with HA-CT and PIP and HA-AQP5 and PIP. Nuclei were labeled with DAPI (blue) and AQP5-PIP interactions are in red (Texas Red). Scale bar, 10µm. Negative controls are shown in Appendix A, Figure A1, left. Images are representative of 3 experiments. (**B**) *Xenopus laevis* oocytes swelling assay. Results are the mean ± S.E.M. (n = 29 for water; n = 24 for Koz-AQP5; n = 28 for HA-AQP5). Statistical analysis was performed using One-way ANOVA; ***: *p* < 0.001. (**C**) The expression of AQP5 was verified by WB in the injected oocytes.

AQP5-PIP interaction was investigated by PLA in the human salivary gland acinar cell line NS-SV-AC co-transfected with HA-AQP5 and PIP plasmids, as their expression was not detectable. NS-SV-AC cells transfected with HA-CT and PIP did not yield a PLA positive signal, whereas the HA-AQP5 and PIP transfected NS-SV-AC cells show a clear PLA signal (Figure 2A). To assess if the HA-tag altered AQP5 function, the functionality of HA-AQP5 was compared to the native Koz-AQP5. To this end, *Xenopus laevis* oocyte swell assay was carried out measuring the osmotic water permeability coefficient (P_f_), following injection of cRNA synthesized from the DNA plasmid constructs. The P_f_ was significantly increased in oocytes injected with Koz-AQP5 (*p* < 0.0001) or HA-AQP5 (*p* < 0.0001) cRNA as compared to oocytes injected with water. The P_f_ value of oocytes injected with HA-AQP5 cRNA was not significantly different (*p* = 0.24) compared to oocytes injected with the Koz-AQP5 cRNA suggesting that the HA-tag did not alter AQP5 functionality (Figure 2B). AQP5 protein expression in oocytes membranes was also confirmed by WB. 28kD bands, corresponding to human AQP5, are identified for each constructs injected and absent in the control water-injected oocytes (Figure 2C).

### 3.2. Molecular Basis for Human AQP5-PIP Protein-Protein Interaction

**Figure 3:**
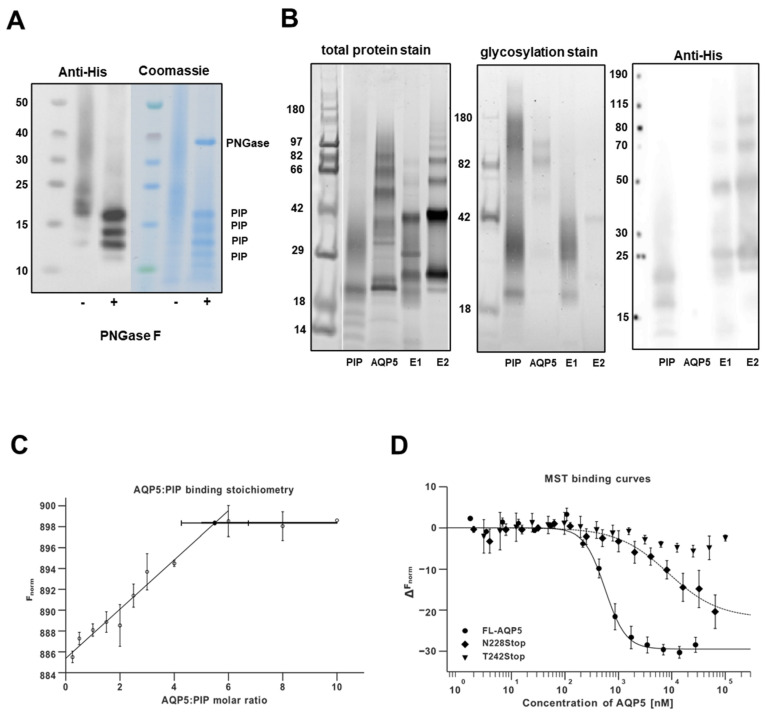
AQP5 and PIP interact at molecular levels. (**A**) WB and SDS-PAGE gel of PIP before and after deglycosylation. (**B**) SDS-PAGE and WB of E1 and E2 samples from the co-elution assay. (**C**) MST binding curves for PIP binding to AQP5 constructs (n = 3). (**D**) Stoichiometry analysis of FL-AQP5 binding to PIP (n = 2). Results are expressed as the mean ± S.E.M.

AQP5-PIP interaction was characterized and quantified at the molecular level using a full-length AQP5, two C-terminal truncated AQP5 constructs (AQP5-N228Stop; AQP5-T242Stop), and a mature C-terminal His-tag PIP, produced in yeast *Pichia pastoris*. As PIP glycosylation may affect its interaction with other proteins, the glycosylation pattern of PIP produced in *Pichia pastoris* was assessed. Before deglycosylation, PIP appeared as a smear (15–25 kDa) with the strongest staining centered at 20kDa in SDS-PAGE and as a smear and two weak bands (13 kDa and 14 kDa) in WB detecting the C-terminal His-tag (Figure 3A). After deglycosylation, the smear disappeared and three strong distinct bands (13 kDa, 14 kDa, and 16 kDa) and an additional weak band (12 kDa) were detected.

The interaction between recombinant PIP and AQP5 was tested using a nickel column co-elution assay with His-tagged PIP bound to the column. Untagged AQP5 was passed through the column and proteins were eluted and analyzed on SDS-PAGE (Figure 3B). In the lane for purified AQP5, protein glycosylation staining revealed multiple bands corresponding to different oligomeric states, typical behavior for AQP5 [3]. A similar pattern observed in both elution fractions (E1, E2) indicated the presence of AQP5. The interaction is further supported by the shift of the ladder bands towards higher molecular weight, suggesting a size increase in the molecule responsible for the laddering. Moreover, AQP5 laddering is visible in the same fractions on the WB stained with anti-His-tag (present on PIP), further supporting an AQP5-PIP interaction. AQP5-PIP interaction was quantified by MST, resulting in a typical binding curve (Figure 3C) which fitted properly to the Hill equation with a dissociation constant (K_d_) of 0.57 ± 0.07 μM. The value of the Hill coefficient, 2.24 ± 0.46, suggests a strong positive binding cooperativity. The MST experiment performed with AQP5-N228Stop or AQP5-T242Stop, lacking the entire C-terminus or retaining the short C-terminal helix that is a known AQP protein-protein interaction site [25,26,27], did not give a binding curve similar to FL-AQP5. For AQP5-N228Stop, the signal decreased slowly without reaching saturation at the highest AQP5 concentrations (Figure 3C) and no reliable K_d_ was obtained (8.67 ± 8.12 μM), suggesting very low affinity or non-specific binding. For AQP5-T242Stop the binding curve was not within the detection limit, suggesting that the AQP5-PIP interaction was abolished. As the FL-AQP5-PIP interaction exhibited positive cooperativity, the stoichiometry of the complex was determined using concentrations of labeled PIP well above the K_d_ (5 μM) and the sample was titrated with FL-AQP5 beyond saturation. This resulted in a kink in the MST data when all binding sites on PIP were occupied and further addition of AQP5 did not increase the F_norm_ (Figure 3B, right). By fitting the data with two linear equations and identifying the intercept, the molar ratio of AQP5:to PIP at the point of saturation yielded 5.49 ± 1.24 (Figure 3D).

### 3.3. Identification of PIP-AQP5 Interaction in Mouse SGs

**Figure 4:**
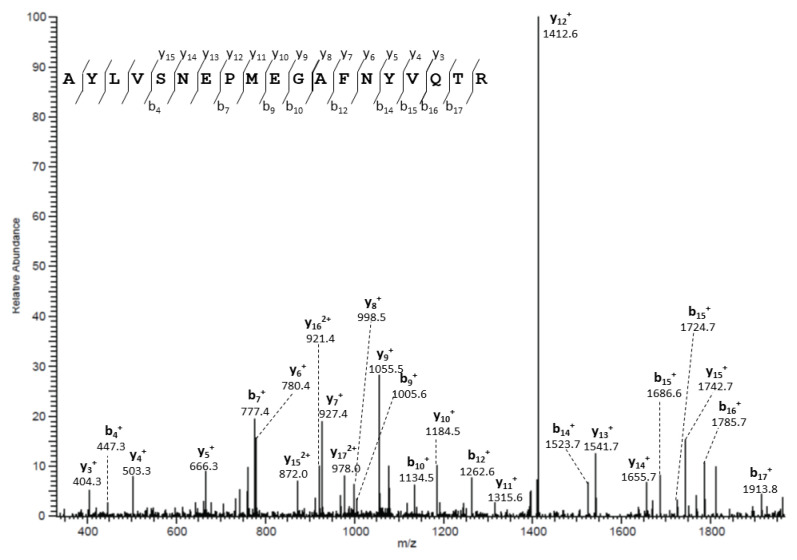
PIP interacts with AQP5 in mouse SGs. Tandem mass spectra of PIP peptides obtained following AQP5 immunoprecipitation in mouse SGs.

PIP was identified as an AQP5 protein partner in mouse SGs by immunoprecipitation using anti-AQP5 antibodies and liquid chromatography/electrospray ionization MS/MS (LC-MS/MS). Indeed, PIP was detected at a low level in some of the experiments. Figure 4 shows a peptide fragment (SNEPMEGAFNYVQ) identified by MS/MS (LC-MS/MS) peak, that corresponds to the mouse PIP protein (NP_032869).

### 3.4. Altered AQP5 Localization in SG of PIP Knockout Mice

**Figure 5:**
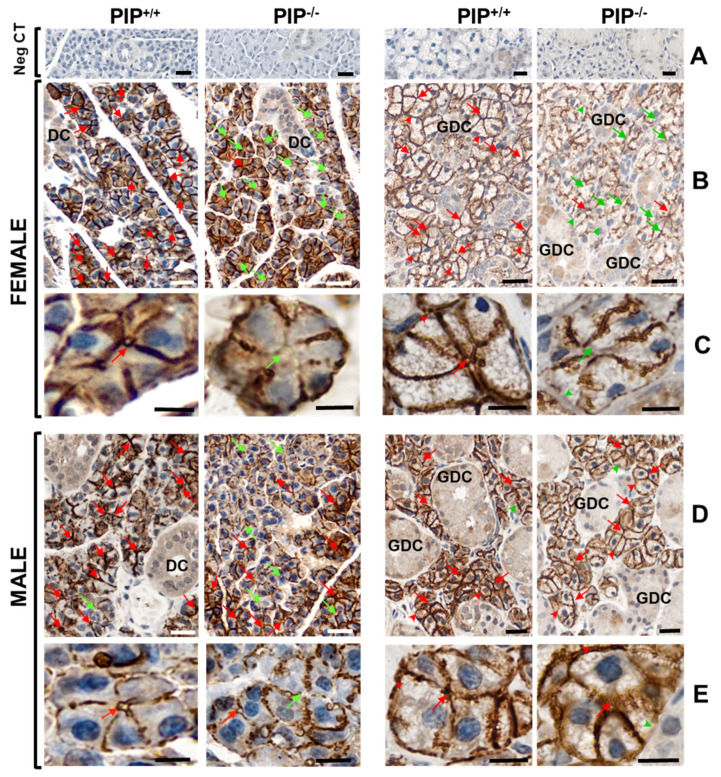
AQP5 localization in P and SM in *PIP^+/+^* and *PIP^−/−^* male and female mice. Negative control (Neg CT-up panels) was performed in the absence of a primary antibody. Images are representative of 4 *PIP^+/+^* and 4 *PIP^−/−^* male mice for P glands, 5 mice for other groups. Red and green arrows indicate respectively the presence or absence of apical staining in acinar cells. Scale bar: 20 µm (images in horizontal lanes (**A**,**B**,**D**)) and 10 µm (images in horizontal lanes (**C**–**E**)).

The effect of PIP deletion in PIP knockout mice (PIP^−/−^) on the AQP5 localization was studied in Parotid (P) and Submandibular (SM) glands by immunostaining using female (Figure 5B,C) and male PIP^−/−^ and PIP^+/+^ mice (Figure 5D,E). In P acini, AQP5 was mainly localized at the apical and lateral membranes in PIP^+/+^ mice, while its apical localization was often missing in PIP^−/−^ mice. Interestingly, the lack of AQP5 immunostaining was more frequent in females than in male PIP^−/−^ mice. Moreover, PIP^−/−^ mice often displayed lateral and rarely basal membrane positivity for AQP5 in acinar cells. In P ducts, a diffuse cytoplasmic immunoreactivity for AQP5 was observed in both PIP^+/+^ and PIP^−/−^ mice. In SM, AQP5 was localized at the apical, lateral, and basal membranes of acinar cells and in the cytoplasm of granular ductal cells in PIP^+/+^ mice. AQP5 was often missing in both lateral and basal membranes as well as in the apical membrane of SM acinar cells from female PIP^−/−^ mice. In contrast, no immunostaining difference could be observed in male PIP^−/−^ mice.

### 3.5. Altered Localization of AQP5-PIP Complexes and AQP5 and PIP in Human Minor SG Acini from SS Patients

**Figure 6:**
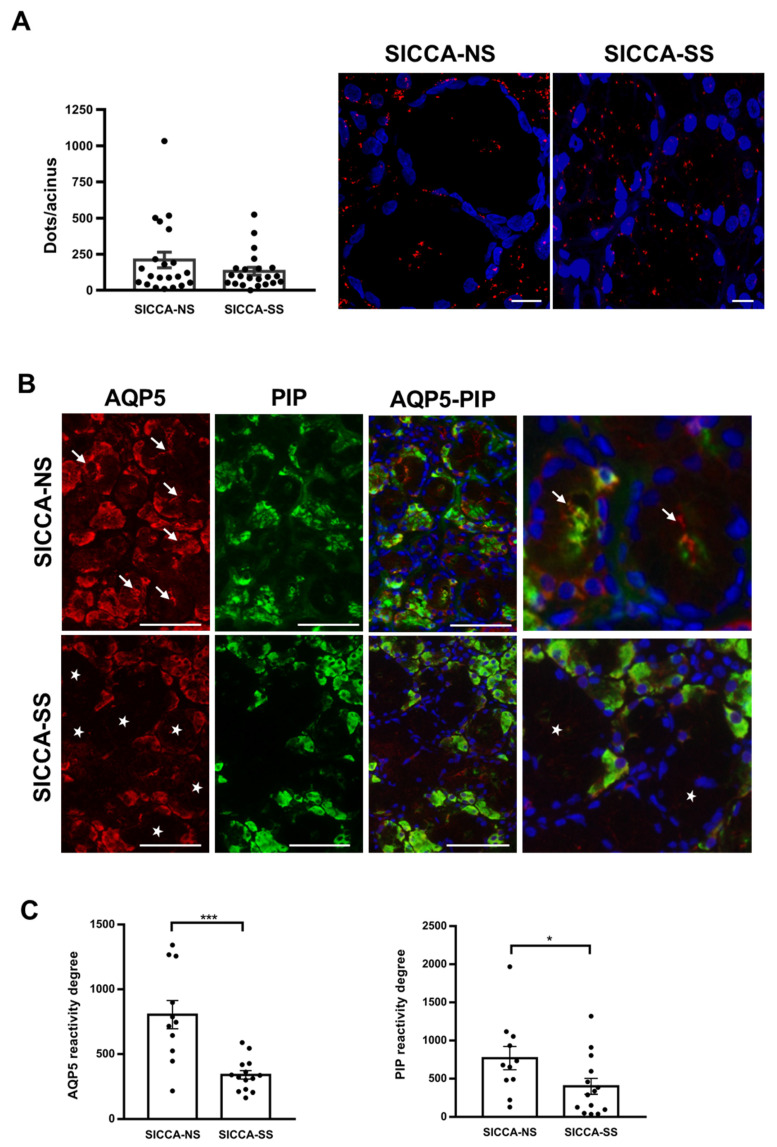
Sjögren’s syndrome alters the expression of AQP5-PIP complexes, and AQP5 and PIP in hMSG. ((**A**) **Left**), quantification of PLA red spots per acinus composed of 15 acinar cells. Results are expressed as the mean ± S.E.M cells (n = 21 SICCA-NS, 22 SICCA-SS). Statistical analysis was performed using Mann Whitney *u* test; *p* > 0.05. ((**A**) **Right**), PLA on hMSG biopsy from SICCA-NS and SICCA-SS. Nuclei were labeled with DAPI (blue) and interactions are represented by red spots. Scale bar, 10 µm. (**B**) Immunofluorescence of AQP5 (red) and PIP (green) in SICCA-NS and SICCA-SS hMSG. Scale bar; 75 µm. Negative controls are shown in Appendix A Figure A1, right. (**C**) Semi-quantitative evaluation of AQP5 and PIP expression. Results are expressed as the mean ± S.E.M. (n = 11 SICCA-NS, n = 14 SICCA-SS). Statistical analysis was performed using Mann Whitney *u* test, * *p* < 0.05; *** *p* < 0.001.

PLA performed on hMSG biopsies obtained from a patient with sicca symptoms and no Sjögren’s syndrome (SICCA-NS) and with sicca symptoms and Sjögren’s syndrome (SICCA-SS) revealed AQP5-PIP complex formation, but there was no significant quantitative difference in the number of complexes (number of red spots) between the two groups (*p* = 0.55). However, the localization of AQP5-PIP complexes was altered in SICCA-SS hMSG. Indeed, in acinar cells, AQP5-PIP complexes were mostly dispersed within the cytoplasm in SICCA-SS hMSG rather than being mainly located at the apical and lateral membranes in SICCA-NS hMSG (Figure 6A). In addition, double immunofluorescence of AQP5 and PIP showed that SICCA-NS hMSG acinar cells displayed strong positive PIP staining (in green) and AQP5 labeling (in red) mainly localized at the apical-luminal membrane and cytoplasm (arrow). In contrast, SICCA-SS hMSG displayed poor PIP expression and AQP5 staining was lacking at the acinar apical membrane (star) (Figure 6B). These observations were corroborated by quantitative fluorescent image analysis for AQP5 (*p* = 0.0003) and PIP (*p* = 0.028) staining between two groups (Figure 6C).

## 4. Discussion

Physiologically, primary saliva is driven by nervous system stimulation, that leads sequentially to intracellular calcium mobilization, chloride secretion via basolateral sodium potassium chloride cotransporter 1 (NKCC1) and apical chloride channel TMEM16/anoctamin-1 (ANO1), sodium passage to the acinar apical lumen, mostly through the paracellular pathway to compensate the negative chloride charge [28].

During this process, AQP5 trafficking to the acinar apical membrane is essential to ensure transcellular water passage driven by the formation of a trans-epithelial osmotic gradient [29,30,31]. Several studies have suggested the involvement of PIP in AQP5 trafficking in mouse LGs and SS pathogenesis [9,11].

Nevertheless, very little is known about the existence of AQP5-PIP complexes in human SGs, the nature of such interaction, its role in SG function, and its possible involvement in the altered AQP5 localization observed in SS hMSG. Based on existing data, we hypothesize that protein complexes made of AQP5, PIP, and other interacting protein(s) could play a role in AQP5 trafficking.

This study aims to shed light on these important aspects by studying the human AQP5-PIP interaction in different models. In vitro, PLA performed on NS-SV-AC cells transfected with HA-AQP5 and PIP plasmids showed the existence of their interaction. The HA-tag had no impact on AQP5 function as the HA-AQP5 and Koz-AQP5 displayed comparable P_f_ in oocyte swelling test, significantly higher than the negative controls and comparable to the reported human and rat AQP5 [32].

The molecular and structural basis of AQP5-PIP interaction was further established using recombinant proteins expressed in yeast *Pichia pastoris*. First, the glycosylation status of the recombinant secreted PIP was analyzed, as glycosylation plays a role in protein structure and function [33]. Recombinant PIP was non homogeneously glycosylated, and the glycan could be removed by deglycosylation. Following deglycosylation, the presence of several PIP bands on both SDS-PAGE and WB were observed. This might result from an ineffective cleavage of the α-factor secretion signaling sequence during overexpression [34]. Moreover, the secretion signal itself contains glycosylation sites that are important for correct protein translocation [35] and probably also exhibits micro-heterogeneity. If not efficiently cleaved during protein maturation, α-factor could contribute to the unclear protein size. The specific role of the glycans in PIP-protein interactions remains unclear. Tissue-specific differences in PIP glycosylation could affect its affinity towards binding partners [17,36]. However, MST data suggest that glycosylation is not likely to play a role in the AQP5-PIP interaction as we observed a sharp transition rather than a gradual slope (in case of micro-heterogeneity of glycosylation) in the MST binding curve for FL-AQP5.

The AQP5-PIP binding curve data fitted to the Hill equation displayed a K_d_ of 0.57 ± 0.07 μM that is much weaker than the PIP-CD4 receptor interaction (K_d_ of 6 nM) [37]. This difference in affinity between proteins is not surprising as protein-protein interactions may involve distinct binding modes specific to the structural nature of either AQP5 (α-helical membrane protein) or CD4 (with several β-sheet immunoglobulin domains). PIP binding to a variety of proteins with different affinities supports its ability to bind its targets in different ways. MST stoichiometry data suggest that on average one molecule of PIP binds 5.5 monomers of AQP5. However, given the large uncertainty in the intercept and the tetrameric structure of AQP5, the most likely state is a 1:4 PIP:AQP5 complex ratio. Despite the difficulty to assess whether PIP binds one or several AQP5 monomers within the tetramer, the positive cooperativity of the binding suggests the presence of more than one AQP5 binding to one PIP. Structurally, it is easy to imagine several C-termini of AQP5 binding to the same PIP as observed for AQP0-calmodulin interaction [26]. In addition, our binding studies suggest that the C-terminal helix of AQP5 does not play a major role in the AQP5-PIP interaction on its own as the partially truncated construct that retains the C-terminal helix (T242Stop) does not bind PIP. We, therefore, hypothesize that the PIP binding site is located after the C-terminal helix, in the distal part of the C-terminus. This part of the C-terminus is likely to adopt a disordered structure as inferred by it being unresolved in the crystal structure of AQP5 [3]. The hypothesis of a PIP-binding site located in the distal part of the C-terminus is supported by PIP pulldown from LGs homogenate using an AQP5 C-terminal peptide consisting of residues 251–265) [9]. Future experiments including crosslinking mass spectrometry, mutational analysis, or structural determination will provide more insight into the structure of the AQP5-PIP complex.

To support the in vitro data, we studied the interaction in mice. In agreement with previous data obtained in mouse LGs [9], AQP5-PIP interaction in mouse SGs was also observed using native protein AQP5-immunoprecipitation followed by proteomic analysis. Following AQP5 immunoprecipitation, PIP could not be detected under our experimental conditions even though the anti-PIP antibodies were capable to detect PIP expression on mouse salivary gland total protein extracts. The lower sensitivity of this method, as compared to proteomic analysis, may account for these data. Besides, no PIP immunoprecipitation followed by AQP5 detection could be achieved due to the lack of ability of commercially available anti-PIP antibodies to immunoprecipitate PIP. The much lower affinity of PIP for AQP5 (±600 nM) i.e., 100 fold lower than the described PIP-CD4 receptor interaction (6 nM), may explain, why in vivo (versus the in vitro fixed cell models), protein-protein affinity hampered consistent recovery after immunoprecipitation and MS detection in a subset of experiments. Nevertheless, PIP was detected unambiguously, as clearly exhibited by the data.

To understand the role of the PIP on AQP5 cellular localization in vivo, we studied the impact of PIP deletion in knockout mice (PIP^−/−^). In PIP^−/−^ mice P and SM acinar cells, AQP5 was lost at the apical and lateral poles compared to the PIP^+/+^ mice. Moreover, the aberrant AQP5 localization was observed essentially in the female mice. Interestingly, in humans, this gender difference is observed as well in SS patients, where 9/10 are women. This may reflect differential mechanisms of protein-protein interactions, that may involve hormonal cues such as estrogens and androgens [1]. Indeed, reduced levels of estrogens have been observed to be responsible for the strong female incidence of SS while androgens suppress the inflammation [38]. In addition, AQP5 and other AQPs can be regulated by sex hormones [39,40,41,42].

AQP5 and PIP localization and interaction were then studied in hMSG from SICCA-NS and SICCA-SS patients by PLA and double immunofluorescence. Quantitative analysis of PLA data indicated that the number of AQP5-PIP interactions in SICCA-NS and SICCA-SS hMSG acini was not significantly different. However, the distribution of the AQP5-PIP complexes in SICCA-SS hMSG acini (mostly cytoplasmic) was predominantly altered compared to SICCA-NS (mainly at the apical plasma membrane). Furthermore, double immunofluorescence showed that the AQP5 labeling at the acinar apical plasma membrane of hMSG appears to be related to the PIP labeling in SICCA-NS (strong labeling) and SICCA-SS (poor labeling). Our data agree with a previous study showing a significant decrease in PIP levels in hMSG, and saliva from SICCA-SS patients [11]. As such, our data suggest that a decrease in PIP expression could account for AQP5 mislocalization in SS SGs [7] and LGs [6].

Based on the data acquired in this study, PIP may be involved in AQP5 trafficking possibly through binding to the cytoskeleton and membrane vesicle mobilization. Indeed, PIP binding to actin [39], myosin, and tropomyosin [43] may serve as an adaptor between AQP5 and actin during the trafficking process, especially considering that actin and tropomyosin have been shown to be involved in the trafficking of AQP2, indeed the latter shares high sequence homology with AQP5 [33]. The reduced expression of PIP observed in SS might interfere with the cytoskeleton-dependent trafficking machinery, resulting in the reduced AQP5 localization observed at the SG acinar apical membrane.

In this work, we show for the first time the existence of an interaction between the human AQP5 and PIP proteins in vitro and in situ, and that altered localization of both AQP5-PIP complexes and AQP5 in SICCA-SS hMSG might be related to decreased PIP expression.

Furthermore, we have determined the structural nature of interaction that involves a fundamental role for the AQP5 C-terminal region. We also determined the stoichiometry of the AQP5-PIP interaction that is compliant with the tetrameric nature of functional AQP5.

## 5. Conclusions

The significance of our results goes beyond the field of salivary gland cell biology and SS pathogenesis. Indeed, considering AQP5 and PIP have been involved in lung and breast cancers [44,45,46,47,48,49], our findings concerning AQP5-PIP protein-protein interaction may help to deepen the comprehension of the pathogenesis in various human pathologies.

## Figures and Tables

**Figure A1:**
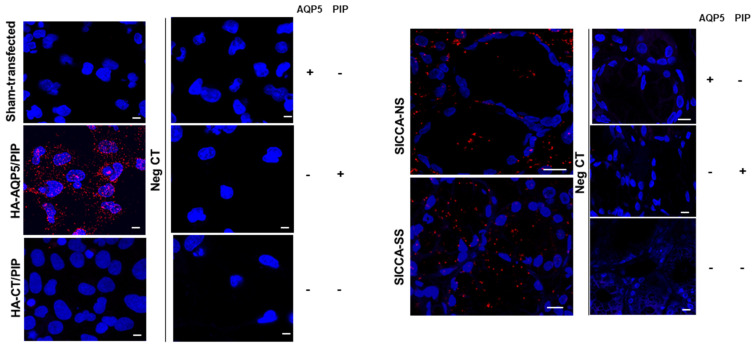
**Protein-protein interactions in transfected NS-SV-AC cells and hMSG.** Protein-protein interactions visualized as red fluorescent spots on transfected NS-SV-AC (**left**) and hMSG (**right**). Nuclei were labelled with DAPI (blue). **Left**, negative controls were performed using NS-SV-AC transfected with HA-AQP5 and PIP in the absence of anti-HA antibody, anti-PIP or both primary antibodies. **Right**, negative controls were performed using hMSG from SICCA-NS in the absence of anti-AQP5 antibody, anti-PIP or both primary antibodies. Scale bar, 10 µm.

## Data Availability

The data presented in this study are available on request from the corresponding author.
